# Anesthesia Analysis of Compound Lidocaine Cream Alone in Adult Male Device-Assisted Circumcision

**DOI:** 10.3390/jcm12093121

**Published:** 2023-04-25

**Authors:** Zhihuan Zheng, Ke Ding, Zhengyan Tang, Ziqiang Wu, Zhongyi Li, Guilin Wang, Benyi Fan, Zhao Wang

**Affiliations:** 1 Department of Urology, Xiangya Hospital, Central South University, Changsha 410008, China; 2 Provincial Laboratory for Diagnosis and Treatment of Genitourinary System Disease, Changsha 410008, China

**Keywords:** anesthetic effect, compound lidocaine cream, adult male, device-assisted circumcision

## Abstract

Objective: to evaluate the anesthetic effect among adult male patients with the single use of compound lidocaine cream in device-assisted circumcision, hoping to provide an anesthetic method for the simplification of the surgical process. Methods: Male adult patients undergoing device-assisted circumcision through prepuce local anesthesia using lidocaine cream in Xiangya Hospital of Central South University from December 2020 to August 2021 were selected. According to different age groups and different surgical procedures, the anesthetic effect of compound lidocaine cream was analyzed considering the aspects of anesthetic cost, anesthetic time, anesthetic duration, anesthetic effect, anesthetic side effects and anesthetic satisfaction. Results: In the study, 99.1% of 649 patients needed only 1 application of compound lidocaine cream to complete the operation. The time taken for anesthesia was short; the whole anesthesia process only required approximately 2–5 min. However, for patients with severe phimosis, the time to complete the anesthesia procedure was correspondingly longer. The pain degree caused by anesthesia was low, and the patients with a pain score of ≤3 points accounted for 96.7%. The anesthetic effect lasted for a sufficiently long period, and the time of algesia recovery from local anesthesia was almost 1 h after surgery. The anesthesia effect was sufficient, and patients with an intraoperative pain score of ≤3 accounted for 98.7%, which could meet the surgical requirements. There were few side effects of the anesthesia. The overwhelming majority of patients were pleased with the anesthesia, and 98.9% of patients had an anesthesia satisfaction score of ≥7. Conclusion: The compound lidocaine cream, as a local anesthetic, is safe and effective for most adult male device-assisted circumcisions. More useful information needs to be corroborated by more advanced evidence, especially for severe phimosis.

## 1. Introduction

Circumcision is a long-standing and widely performed urologic surgery [1]. Phimosis will affect the normal development of the penis head, lead to repeated inflammation of the prepuce and urethral mouth and increase the probability of HIV infection and even the occurrence of penile cancer [2]. In addition to medical indications, religious and cultural factors, personal preferences or the need for medical aesthetic quality, it is estimated that one third of men around the world have undergone circumcision [3]. According to a survey regarding the foreskin of boys in China, the phimosis incidence decreases gradually with age; however, it is still as high as 6.81% at puberty [4]. Because many parents do not have a clear understanding of the disease, many boys are not treated in a timely manner. When these boys become adults, they are aware of the problems associated with redundant prepuce, such as repeated genitourinary–genital inflammation in themselves and their sex partners and the risk of HIV or HPV infection. For adult males aged 20–35, who are at the age with the greatest risk of HIV infection, circumcision can reduce the likelihood of HIV infection [5,6,7]. To maintain their own health or to meet the requirements of their sexual partners, these adult males with phimosis have a quite strong incentive to undergo surgical treatment. Barriers to the demand for and uptake of circumcision have been well documented. Studies have already shown that the fear of pain during circumcision is the most frequent deterrent regarding the uptake of circumcision [8,9,10]. Dorsal penile nerve block (DPNB) anesthesia is commonly used in traditional circumcision, because of its definite intraoperative and postoperative analgesia effects. However, the DPNB procedure is slightly more complicated. In addition, many patients have a poor experience at the time when the needle carrying the narcotic penetrates the skin, although this will soon pass. Some patients even express concerns about whether it will damage their sexual function. With the innovation of technology, device-assisted circumcision has gradually been promoted. These novel devices may help clinicians to shorten the surgical time and reduce sutures [11]. The requirement for the duration of local anesthesia is also reduced. Therefore, we hope to explore a safe, effective, simple and needle-free anesthetic method for device-assisted adult circumcision and further simplify the surgical procedure.

A eutectic mixture of local anesthetics (EMLA) is a mixture of anesthetics such as lidocaine and prilocaine, suspended in an oil-in-water emulsion [12]. The high concentration of the local anesthetics (LA) stimulates the transdermal spread of the active ingredients, providing effective surface analgesia on intact skin. Over the past few years, a large body of clinical data has been amassed demonstrating the clear superiority of EMLA over a placebo in reducing the acute pain inflicted by a wide variety of medical/surgical procedures on the superficial skin surface. The safety and efficacy of EMLA in neonatal circumcision have gained considerable recognition [13,14]. There are a few studies on the use of lidocaine cream in device-assisted adult circumcision; however, most of them focus on the evaluation of surgical instruments, such as the Shang ring. To the best of our knowledge, this study is the first to specifically evaluate the anesthetic effects of compound lidocaine cream for device-assisted male circumcision in China.

## 2. Methods

This study is an observational study and has been approved by the Ethics Committee of Xiangya Hospital. Its ethical approval number is 202106106.

### 2.1. Patients

Male patients with the use of only compound lidocaine cream for foreskin surgery anesthesia were enrolled from December 2020 to August 2021 in Xiangya Hospital, Central South University. The anesthetic effect of compound lidocaine cream was analyzed in terms of anesthetic cost, anesthetic time, anesthetic duration, side effects and anesthesia acceptance based on different age stages and different surgical methods.

Inclusion criteria: (1) adult male; (2) undergoing circumcision. Exclusion criteria: (1) age < 18 years old; (2) patients with acute local infection or congenital abnormality of the penis; (3) patients with anesthetic allergies; (4) patients with contraindications, such as coagulation disorders, blood diseases and heart disease.

According to the inclusion and exclusion criteria, a total of 654 patients were screened and enrolled, including 3 patients who could not be contacted and 2 patients who refused follow-up; therefore, 649 patients were finally screened out in the study cohort. According to the World Health Organization criteria for the age stratification of men, we divided the study subjects into 3 groups: 618 cases aged 18–44, 27 cases aged 45–59 and 4 cases aged 60–74.

### 2.2. Surgical Process

**Figure 1:**
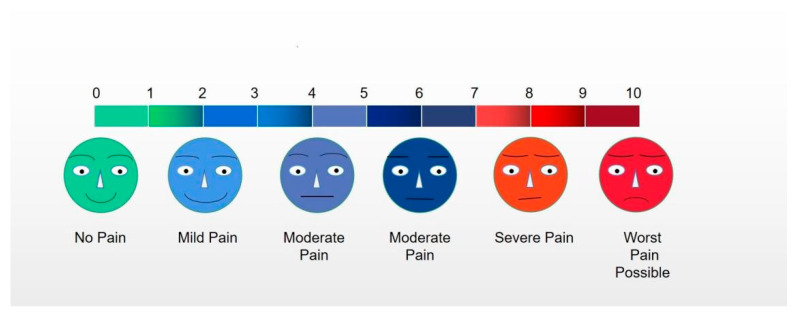
Pain score with VAS. Note: The degree of pain increased with the increase in VAS score.

We used compound lidocaine cream (Tongfang Pharmaceutical Co., Ltd., Beijing, China) alone for anesthesia. This product is a compound preparation consisting of procaine and lidocaine, containing procaine 25 mg and lidocaine 25 mg per gram. First, 5–10 g of compound lidocaine cream was evenly applied to the surface of the prepuce 15–20 min before the operation: at first, the foreskin was turned over with the cooperation of the patient, and the inner foreskin was treated, including the lower frenulum. Then, the foreskin was restored, and the outer foreskin was treated. For patients with true phimosis, we first applied the cream to the surface of the foreskin, gently expanded the gap between the foreskin and the inner plate with the help of instruments and then applied the cream to the inner plate. In patients with excessively severe phimosis, we performed a dorsal incision after topical medication and administered anesthetics to the inner plate. After the application, we evaluated the level of analgesia by the visual analog scale (VAS) [15] and adverse reactions during anesthesia for each patient. Pain scores ranged from 0 to 10, with 0 indicating no pain and 10 indicating extreme pain, as is shown in Figure 1.

The patient chose the surgical device voluntarily: either a prepuce loop ligature (similar to PrePex) or circumcision assisted by a disposable suture device (similar to a no-flip Shang ring). The operation was performed by an experienced surgeon. After anesthesia, the surgeon used a caliper to select the appropriate model of the instrument. Specific surgical procedures for prepuce loop ligature were as follows: The placement ring was left at the base of the penis, and the inner ring was inserted between the glans and the foreskin. The elastic loop was placed on the loop-cutting line. The assistive devices and necrotic prepuce were removed 7 days after surgery. Specific surgical procedures for circumcision assisted by a disposable suture device were as follows: The inner protector was inserted under the prepuce to cover the glans, and the outer ring was then secured to the inner protector; finally, the surgeon initiated the cutting trigger to remove the distal prepuce. The assigned surgeons had performed prepuce loop ligature or circumcision assisted by a disposable suture device more than 200 times and were trained to use the devices before the study began. The training included the choice of device type, pain scoring, surgery skills, adverse reactions to compound lidocaine cream and corresponding treatment measures. During the operation, we observed the patient’s condition, asked the patient about his discomfort and recorded it. After surgery, we evaluated patients’ intraoperative pain peak scores.

### 2.3. Follow-Up

Patients were followed up 4–6 weeks after surgery. We asked patients by phone about pain recovery and adverse reactions to anesthesia within 10 h after surgery and collected their views on anesthesia satisfaction.

There were 4 questions that examined patients’ satisfaction, attitude, the advantages and disadvantages of the combined cream and recorded the proportion of each opinion. The satisfaction survey included the following questions:

1. Are you satisfied with the anesthetic effect of lidocaine combined cream? Please tell us your satisfaction in the form of a score after considering the anesthesia cost, anesthesia method and anesthesia effect. A score of 0 represents very dissatisfied and a score of 10 represents very satisfied. The higher the value, the more satisfied the patient.
2. Are you willing to use lidocaine combined cream as your first choice of anesthesia if you are undergoing circumcision again?
3. What do you think is the greatest advantage of combined lidocaine cream for anesthesia during circumcision?
4. What do you think needs to be improved in the anesthesia of combined lidocaine cream for circumcision?

## 3. Results:

### 3.1. Anesthetic Expenses

Among the 649 patients in this study, 643 (99.1%) patients needed only one application of compound lidocaine cream during the entire operation, and 6 (0.9%) patients were treated with 2 compound lidocaine cream applications.

The cost of the compound lidocaine cream was CNY 49.5 per tablet, and only 1 compound lidocaine cream application was sufficient for most patients. However, a very small number of patients who were sensitive to pain needed another application during surgery. The total anesthetic cost was between CNY 49.5 and 99.

### 3.2. Time and Pain of Anesthesia

#### 3.2.1. Anesthesia Time Cost

The patient was anesthetized as described in the Methods section, and the entire anesthesia process took approximately 2–5 min for normal phimosis. However, for patients with severe phimosis, the time to complete the anesthesia procedure was correspondingly longer, taking approximately 10 to 20 min due to the gentle operation and additional steps.

#### 3.2.2. Anesthesia Pain Degree

The VAS pain score scale was used to evaluate the anesthesia pain degree for each patient undergoing surgery. During anesthesia, 96.7% of patients had a pain score of 3 or less, whereas patients with severe phimosis had higher VAS scores for anesthesia, as is shown in Table 1.

### 3.3. Duration of Anesthesia

#### 3.3.1. Initial Time of Pain Detection

In this study, a total of 649 patients were followed up for pain evaluation within 10 h after surgery. The follow-up found that as the compound lidocaine cream was absorbed and metabolized, the patient began to experience wound pain. Most patients (75.8%) began to feel pain within 1–2 h after surgery. The maximum painless state lasted for 6 h, as is shown in Table 2.

#### 3.3.2. Age Distribution Characteristics

Among the 649 patients followed up in this study, 618 were 18–44 years old, 27 were 45–59 years old and 4 were 60–74 years old. The initial time for postoperative pain detection varied among different patients, as is shown in Table 3.

### 3.4. Anesthetic Effect

The VAS pain score was used to evaluate the intraoperative anesthesia effect. The lower the pain score, the better the anesthesia. Overall, 98.7% of patients had intraoperative pain peak scores less than or equal to 3, whereas patients with severe phimosis had higher VAS scores for surgery, as is shown in Table 4.

### 3.5. Anesthetic Side Effect

The adverse events of compound lidocaine include headache, dizziness, nausea, vomiting, anaphylaxis (or even anaphylactic shock), blood pressure decline or heart rate drops, chills and transient erythema. In our study, transient erythema was the most common adverse event. However, the erythema may resolve spontaneously after 10–20 min without other serious reactions. Except for transient erythema, five patients showed other anesthetic side effects. The overall incidence of adverse events (except for transient erythema) was extremely low, and the adverse events (such as headache, dizziness, nausea, vomiting and transient erythema) were not harmful, as is shown in Table 5.

### 3.6. Anesthesia Satisfaction

A satisfaction survey for the 649 adult patients that underwent circumcision was conducted. Overall, 97.1% of patients had a satisfaction score of 8 or above; 92.9% of the patients would be willing to use the cream as the first choice for anesthesia if they underwent circumcision again. The advantages of compound lidocaine cream include comfort, the lack of needles and safety. Defects that still need improvement include mild pain, transient itching and the price, as is shown in Table 6.

## 4. Discussion

We found that applying lidocaine–procaine cream to the redundant prepuce reduces the pain of adult circumcision, as measured by VAS pain scores. This is consistent with what Bo-Dong Lv et al. observed in their experiments [16]. Adult men using compound lidocaine cream still experienced mild pain during surgery; however, this level of pain was considered acceptable for patients based on pain scores. However, it is important to note that the use of compound cream to administer local anesthesia in patients with severe phimosis, even with instrumental assistance, needs careful consideration. The increased steps and time required to perform anesthesia offset the advantages of the simplified procedure provided by the application of the cream. These patients tend to have higher VAS scores during anesthesia and during surgery, although there is no significant difference in postoperative anesthesia duration or satisfaction. For such patients, DPNB may be a better choice.

In this study, 643 (99.1%) patients only required one application of compound lidocaine cream during the operation, whereas the remaining 6 (0.9%) patients needed 2 compound lidocaine cream applications to undergo the surgery. Thicker foreskins due to repeated inflammation were observed in these six patients. This may be because the efficacy of lidocaine–procaine cream is affected by the method of application and dose. A thicker foreskin results in the uneven distribution of compound creams, which may cause changes in the tissue concentrations of lidocaine and procaine and in sub-therapeutic anesthetic concentrations in some areas. Another possible reason is that an overly thick prepuce interferes with the surgeon’s operation and prolongates the operation time. For such patients with thick foreskins, we need to adjust the dosage of the cream appropriately. Adult phimosis could be further investigated regarding the method of anesthesia because in phimosis patients, prepuce inflammation and adhesion are severe, and the compound lidocaine cream is unable to penetrate effectively.

Among the 649 patients enrolled, approximately 2 thirds of patients had transient erythema at the site of cream application, with a faint burning sensation. Only five patients had other adverse events due to compound lidocaine cream (except for transient erythema). Moreover, all adverse reactions disappeared approximately 10–30 min after surgery. It could be seen that the use of compound lidocaine cream for anesthesia resulted in few adverse events. Finally, we conducted a patient satisfaction survey; combined with the comprehensive evaluation of the anesthesia cost, anesthesia method and anesthesia effect, the study found that the proportion of patients with scores of ≥7 points was 98.9%. Moreover, 97.6% of the patients were willing to choose compound lidocaine cream for anesthesia if they underwent prepuce surgery again. This indicated that the vast majority of patients were satisfied with this anesthesia method.

The main current analgesic method for circumcision is lidocaine dorsal penile nerve block (DPNB). Studies have shown that anesthesia injection has a better analgesic effect than EMLA. However, anesthetic injections are considered painful and are associated with a risk of systemic toxicity due to the accidental injection of local anesthetics into blood vessels. Penile nerve blocks are not always successful, with a failure rate of 4 to 6.7% [17], meaning that a significant number of patients will require another injection of local anesthesia. In addition, we found that adult male patients were concerned about the adverse effects of local anesthesia on their sexual function, which may be related to the pain and fear associated with needle insertion. Although there is no evidence to support the idea that local injection anesthesia can impair sexual function, these psychological changes deserve more attention. Ozen, V et al. proposed that ultrasound-guided dorsal penile nerve block with the in-plane technique is a safe method for regional anesthesia and can provide effective postoperative analgesia for male circumcision [18]. DPNB-related complications can be prevented with real-time imaging provided by an ultrasound. However, the use of an ultrasound during surgery is more expensive and places higher technical requirements on the surgeon, which complicates the whole procedure. EMLA is patient-friendly as it eliminates needle fear, anxiety and injection pain at the beginning of the procedure. Compound lidocaine cream is also a more manageable anesthetic option for surgeons.

Kostis I. Gyftopoulos et al. mentioned in their study that the anesthesia effect of EMLA is insufficient to support the whole procedure of circumcision [19]. We believe that this is due to the different methods of surgery. The procedure in this study was limited and did not include traditional circumcision by hand, because compound creams require as short an operation as possible. Thanks to technological advances in ligating rings and suture devices, the time required for circumcision has been greatly reduced, paving the way for the use of compound creams for anesthesia.

Another limitation of this study is the small number of elderly patients included. In our cohort, there were only four elderly patients. It is thus difficult to draw a convincing conclusion. Older people do not pay sufficient attention to phimosis. The risk of anesthesia is known to be higher in older people. The safety and efficacy of compound lidocaine cream in the elderly population still needs to be further verified.

The use of compound lidocaine cream in adult male circumcision has not yet become mainstream. However, the anesthetic effect of compound lidocaine cream in pediatric prepuce surgery has been confirmed in some studies. Mujeeb, S. et al. suggest that EMLA is comparable to pudendal nerve block for anesthesia and analgesia [20]. Taddio, A. et al. agree that the lidocaine–prilocaine cream is efficacious and safe for the prevention of pain from circumcision in neonates [13]. It is reasonable for us to believe that the use of lidocaine cream as an anesthetic for adult male device-assisted circumcision will become more common with the spread of ligating rings and suture devices. The lidocaine–prilocaine cream poses little risk to adult males and blocks afferent nociceptive input for several hours after application. This study was a single-center observational study without a control group. Therefore, more studies and evidence are needed to determine whether compound lidocaine cream can be the first anesthesia choice for adult male device-assisted circumcision.

## 5. Conclusions

In conclusion, lidocaine–procaine cream, as a local anesthetic, is safe and effective for adult male device-assisted circumcision. If the patient chooses a prepuce loop ligature or circumcision assisted by a disposable suture device, compound lidocaine cream provides a relatively easy alternative to nerve blocks. We recommend that it be routinely considered in adult men who undergo this procedure to reduce their pain. However, whether lidocaine cream can completely replace local injection anesthesia and become the mainstream anesthesia method in adult prepuce surgery with device-assistance still needs further research and high-level evidence, especially for severe phimosis.

## Figures and Tables

**Table 1:** VAS Anesthesia Pain Score Scale.

VAS Pain Score	0	1	2	3	4	5	6	7	8	9	10	Total
Phimosis (percent, %)	289 (44.53)	207 (31.89)	88 (13.56)	21 (3.24)	4 (0.62)	2 (0.31)	3 (0.46)	1 (0.15)	0	0	0	615 (94.76)
Severe Phimosis (percent, %)	0	5 (0.77)	8 (1.23)	10 (1.54)	5 (0.77)	2 (0.31)	2 (0.31)	2 (0.31)	0	0	0	34 (5.24)

Note: % represents the percentage of all 649 patients.

**Table 2:** Postoperative pain summary.

The Initial Time of Pain Detection	No Change	1 h after Surgery	2 h after Surgery	3 h after Surgery	4 h after Surgery	5 h after Surgery	6 h after Surgery
Number	14	135	357	81	37	16	9
Percentage (%)	2.1	20.8	55	12.5	5.7	2.5	1.4

**Table 3:** Postoperative pain in patients aged 18–44/45–59/60–74 years.

The Initial Time of Pain Detection	No Change	1 h after Surgery	2 h after Surgery	3 h after Surgery	4 h after Surgery	5 h after Surgery	6 h after Surgery	Total
Number aged 18–44 years	10	129	343	78	35	15	8	618
Percentage (%)	1.6	20.8	55.5	12.6	5.6	2.6	1.3	100
Number aged 45–59 years	3	6	13	2	2	0	1	27
Percentage (%)	11.1	22.2	48.2	7.4	7.4	0	3.7	100
Number aged 60–74 years	1	0	1	1	0	1	0	4
Percentage (%)	25	0	25	0	25	25	0	100

**Table 4:** Intraoperative VAS pain score.

VAS Pain Score	0	1	2	3	4	5	6	7	8	9	10	Total
Phimosis (%)	252 (38.83)	213 (32.82)	135 (20.80)	8 (1.23)	5 (0.77)	1 (0.15)	1 (0.15)	0	0	0	0	615 (94.76)
Severe Phimosis (%)	4 (0.62)	8 (1.23)	14 (2.16)	6 (0.92)	1 (0.15)	1 (0.15)	0	0	0	0	0	34 (5.24)

**Table 5:** Summary of anesthesia side effects for the combined cream.

Side Effects	Headache and Dizziness	Nausea and Vomiting	Blood Pressure Decline or Heart Rate Drops	Chills	Transient Erythema
Number	3	2	0	0	431
Percentage (%)	0.5	0.3	0	0	66.4

**Table 6:** Anesthesia satisfaction survey.

Questions Asked	Evaluation and Description	Number	Percentage (%)
Question 1 (Satisfaction Scores)	0–3	0	0
	4–7	19	2.9
	8–10	630	97.1
Question 2 (Attitudes)	Very willing	603	92.9
	Somewhat willing	41	6.3
	Not willing	5	0.8
Question 3 (Advantages)	Comfortable	141	21.7
	No needle	386	59.5
	Safe	96	14.8
	Else	26	4.0
Question 4 (Shortcomings)	Mild pain	272	42.0
	Transient itching	189	29.1
	Price	107	16.5
	Else	81	12.4

## Data Availability

The raw data supporting the conclusions of this article will be made available by the authors, without undue reservation.
